# Exploring the value of genomic predictions to simultaneously improve production potential and resilience of farmed animals

**DOI:** 10.3389/fgene.2023.1127530

**Published:** 2023-05-12

**Authors:** Masoud Ghaderi Zefreh, Andrea B. Doeschl-Wilson, Valentina Riggio, Oswald Matika, Ricardo Pong-Wong

**Affiliations:** ^1^ The Roslin Institute and R(D)SVS, University of Edinburgh, Edinburgh, United Kingdom; ^2^ Centre for Tropical Livestock Genetics and Health (CTLGH), The Roslin Institute and R(D)SVS, University of Edinburgh, Edinburgh, United Kingdom

**Keywords:** resilience, robustness, reaction norm, genomic prediction, genomic selection, GxE, trade-off

## Abstract

Sustainable livestock production requires that animals have a high production potential but are also highly resilient to environmental challenges. The first step to simultaneously improve these traits through genetic selection is to accurately predict their genetic merit. In this paper, we used simulations of sheep populations to assess the effect of genomic data, different genetic evaluation models and phenotyping strategies on prediction accuracies and bias for production potential and resilience. In addition, we also assessed the effect of different selection strategies on the improvement of these traits. Results show that estimation of both traits greatly benefits from taking repeated measurements and from using genomic information. However, the prediction accuracy for production potential is compromised, and resilience estimates tends to be upwards biased, when families are clustered in groups even when genomic information is used. The prediction accuracy was also found to be lower for both traits, resilience and production potential, when the environment challenge levels are unknown. Nevertheless, we observe that genetic gain in both traits can be achieved even in the case of unknown environmental challenge, when families are distributed across a large range of environments. Simultaneous genetic improvement in both traits however greatly benefits from the use of genomic evaluation, reaction norm models and phenotyping in a wide range of environments. Using models without the reaction norm in scenarios where there is a trade-off between resilience and production potential, and phenotypes are collected from a narrow range of environments may result in a loss for one trait. The study demonstrates that genomic selection coupled with reaction-norm models offers great opportunities to simultaneously improve productivity and resilience of farmed animals even in the case of a trade-off.

## 1 Introduction

Farmed animals can be exposed to a wide range of environmental challenges during their development and lifespan. Climate change is known to exacerbate these challenges, e.g., through increased temperature fluctuations or more frequent occurrence of extreme weather conditions (Thornton et al., 2007; Baumgard et al., 2012) and associated food shortages or increased burden of infectious pathogens (Tomley and Shirley, 2009). It is thus desirable, for both animals’ welfare and productivity, that animals not only have a high production potential in ideal environmental conditions, but are also able to maintain it when exposed to environmental or infectious challenges. In the animal breeding community, this capacity has been defined as resilience and has been considered as an important breeding goal since decades (Bisset and Morris, 1996; Hermesch and Dominik, 2014; Knap and Doeschl-Wilson, 2020). In the context of intensive livestock production, resilience and robustness are often used interchangeably (Colditz and Hine, 2016; Friggens et al., 2016). Here, we follow the definition of Knap (2005), who defined robustness as the combination of high production potential with high resilience to external stressors, allowing for unproblematic expression of that production potential in a wide variety of environmental conditions. The performance of an animal in an environment is thus determined by its production potential, resilience, and the level of the overall challenge the animal faces in that environment.

Neither the resilience nor the production potential of an animal in ideal environmental conditions are directly measurable. Instead, estimates for both traits can be obtained by using so called reaction norm (RN) models in which the performance of an animal is regressed against the environmental challenge level (de Jong, 1990). In the case of a linear RN model, the production potential then refers to the model intercept and resilience refers to the inverse of the slope (Mulder, 2016). Genetic parameter estimates and estimated breeding values for both traits can then be obtained through random regression approaches (Strandberg, 2006). Such random regression models have been used to estimate animals’ resilience to temperature or other quantifiable climatic measures (Knap and Su, 2008; Nguyen et al., 2016; Bohlouli et al., 2019; Sánchez-Molano et al., 2019).

One of the hurdles for estimating resilience is that in many cases the environmental challenge level is unknown or difficult to quantify. This is particularly the case when animals are exposed to infectious pathogens (Knap and Doeschl-Wilson, 2020) or a whole cocktail of different environmental stressors (e.g., of multiple pathogens, sub-optimal nutritional resources, etc.). In these cases, the common approach is to use the contemporary group mean performance as a proxy for the environmental challenge level in the RN models (Knap, 2005; Strandberg, 2006; Rashidi et al., 2014; Hermesch et al., 2015). To avoid bias in the genetic parameter estimates for the regression parameters, the contemporary group means (e.g., herd effect or herd-season-year effect) representing the environmental value in the regression models are usually estimated together with the regression parameter estimates in an iterative procedure (Calus et al., 2004; Knap and Su, 2008). Given that the average group performance is most likely a combination of many factors, this proxy may indeed provide a good overall description of the type of environment the animals are exposed to (Strandberg, 2006). However, it is important to note that this measure is only an approximation of the true environmental challenge that each individual is exposed to, and that the ideal environment and thus also the deviation from it, i.e., the challenge level, may vary between individuals. However, it is currently not known how inaccuracies in the estimates of the actual environmental challenge levels of individuals affect the prediction accuracies for production potential and resilience.

Another obstacle in estimating resilience through a RN approach is the lack of performance data of an individual across multiple environments. Many phenotypes have limited measurements per animal, e.g., carcass weight and hence rely on phenotypic records of related individuals (Strandberg, 2006). The sparsity of phenotypic records for an individual can have a negative impact on the accuracy of estimated breeding values (EBVs) (Cameron, 1997). However, genomic prediction has proven to be beneficial in improving prediction accuracies under these conditions (Meuwissen et al., 2001; Mulder, 2016), with a few studies having assessed the benefit of genomic prediction on the accuracies of resilience and production potential. In particular, Calus et al. (2004) showed that prediction accuracies of RN model parameters strongly depend on how related individuals are distributed across different environments. Furthermore, in scenarios where related individuals are reared in the same environment, accuracy of EBVs for RN slope and intercept are adversely affected (Calus et al., 2004). However, it is not known whether genomic prediction can partly or completely overcome this issue, because the genomic relationship matrix accounts for similarities between related as well as unrelated individuals.

Numerous studies have applied RN models to predict animals’ resilience or performance under different environmental challenge conditions (Knap and Su, 2008; Herrero-Medrano et al., 2015; Li and Hermesch, 2016; Oliveira et al., 2019; Song et al., 2020; Garcia-Baccino et al., 2021). One of the clear benefits of linear RN models is that they provide genetic parameter estimates for both breeding goal traits, production potential and resilience. Therefore, depending on the genetic parameters, it may be possible to concomitantly select for both traits given appropriate indices. However, RN models are also known to require a large amount of data for convergence (Knap and Su, 2008). Therefore, many studies continue to use conventional genetic evaluation models for production performance, that don’t explicitly quantify the environmental challenge level, but account for potential differences in these by fitting fixed group effects. Although few studies have assessed selection response in performance in different environments for RN models compared to conventional models (Kolmodin and Bijma, 2004; Mulder, 2016; Mulder and Rashidi, 2017), the genetic improvement in production potential and resilience as breeding goal traits, that can be achieved by either approach, has not been explicitly assessed. These are likely to also depend on the environmental conditions under which phenotypic performance records are collected (Calus et al., 2004; Mulder, 2016; Le et al., 2022).

Therefore, the first objective of this study was to assess the benefits of using genomics over pedigree-based predictions in obtaining accurate and unbiased breeding value estimates for production potential and resilience from linear RN models. In particular, we assessed how prediction accuracies depend on the number of phenotypic records per individual, the distribution of related animals across environments, and on the ability to accurately quantify the environmental challenge level. The second objective was to assess the feasibility of RN models to improve production potential and resilience and compare these to the response to selection that can be achieved by using conventional models under a range of conditions.

## 2 Methods

The potential benefit of genomic selection to select for production potential and resilience under a linear RN model was assessed using simulations of an outbred population of farmed animals. Without loss of generality, in this study the genomes of sheep populations were simulated, but it is expected that results would be similar for other population of terrestrial farmed animals. To assess the impact of genomics and other factors on prediction accuracies and bias, a sheep population undergoing random selection was simulated. To assess response to selection, a population undergoing selection for 10 generations under different selection strategies was simulated. Each simulation scenario was replicated 100 times.

### 2.1 Genetic models for production potential and resilience

The phenotype was defined assuming a RN model, where the individual’s performance is affected by two components, simulated as random effects: one related to the performance under ideal condition and another being linearly related to an environmental challenge level the individual is exposed to.

Here, the respective components are denoted as production potential (subscripts 0) and resilience (subscripts *R*) and both are assumed to be under genetic (*A*) and environmental (*E*) control. Hence, the performance, *y*, of individual *j* in an environment with challenge level *X* is:

$$y_{j,k}\left(X\right)=\left(\mu_{0}+A_{0_{j}}+E_{0_{j,k}}\right)+X\left(\mu_{R}+A_{R_{j}}+E_{R_{j,k}}\right),$$ (1)

where the subscript *k* corresponds to the time of measurement in case of multiple records for an individual. In the above equation, *μ*_0_ refers to the population mean performance in the absence of challenge, *μ*_*R*_ is the population average rate of reduction in performance (as the environmental challenge generally has adverse effect on performance, *μ*_*R*_ is negative). The terms $A_{0_{j}}$ and $A_{R_{j}}$ are the additive genetic effects for production potential and resilience and $E_{0_{j,k}}$ and $E_{R_{j,k}}$ are their environmental deviations from the following normal distributions

$$\left(\begin{array}{c} A_{0_{j}}\\ A_{R_{j}} \end{array}\right)\in N\left(0,\left(\begin{array}{cc} \sigma_{A_{0}}^{2} & \sigma_{A_{0}A_{R}}\\ \sigma_{A_{0}A_{R}} & \sigma_{A_{R}}^{2} \end{array}\right)\right),\;\left(\begin{array}{c} E_{0_{j,k}}\\ E_{R_{j,k}} \end{array}\right)\in N\left(0,\left(\begin{array}{cc} \sigma_{E_{0}}^{2} & 0\\ 0 & \sigma_{E_{R}}^{2} \end{array}\right)\right),$$ (2)

where $\sigma_{A_{0}}^{2}$ , $\sigma_{A_{R}}^{2}$ and $\sigma_{A_{0}A_{R}}^{2}$ are the genetic variance of production potential, resilience and the genetic covariance and $\sigma_{E_{0}}^{2}$ and $\sigma_{E_{R}}^{2}$ are the environmental variance of production potential and resilience, respectively.

With the parameterisation in Eq. 1, the heritabilities of the production potential and resilience are

$$h_{0}^{2}=\frac{\sigma_{A_{0}}^{2}}{\sigma_{A_{0}}^{2}+\sigma_{E_{0}}^{2}},\;h_{R}^{2}=\frac{\sigma_{A_{R}}^{2}}{\sigma_{A_{R}}^{2}+\sigma_{E_{R}}^{2}}.$$ (3)

The heritability of the performance at a given environmental challenge level *X* follows (Kolmodin and Bijma, 2004)

$$h^{2}\left(X;\sigma^{2}\right)=\frac{\sigma_{A_{0}}^{2}+X^{2}\sigma_{A_{R}}^{2}+2X\sigma_{A_{0}A_{R}}}{\sigma_{A_{0}}^{2}+X^{2}\sigma_{A_{R}}^{2}+2X\sigma_{A_{0}A_{R}}+\sigma_{E_{0}}^{2}+X^{2}\sigma_{E_{0}}^{2}}.$$ (4)

We used low heritability values (0.1) for $h_{0}^{2}$ and $h_{0}^{2}$ (see Table 1), however, the profile of changes with respect to other parameters did not change for other heritabilities (result shown in Supplementary Table SA1).

**TABLE 1:** Parameter values used in simulating phenotypes.

Parameter	Value	Alternative values
*μ*_0_	10	—
*μ*_*R*_	−3	—
$\sigma_{A_{0}}^{2}$	0.1	0.3, 0.6
$\sigma_{A_{R}}^{2}$	0.1	0.3, 0.6
$\sigma_{E_{0}}^{2}$	0.9	0.7, 0.4
$\sigma_{E_{R}}^{2}$	0.9	0.7, 0.4
$\rho_{A_{0}A_{R}}$	−0.5	0, 0.5
*F*_*N*_	10	—
*ϵ*	0.1	0.2, 0.3, 0.5
Range of *X*	[0,2]	[0, 1], [1, 2]

*μ*_0_: mean production potential, *μ*_*R*_: mean resilience, $\sigma_{A_{0}}^{2}$ : additive genetic variance for production potential, $\sigma_{A_{R}}^{2}$ : additive genetic variance for resilience, $\sigma_{E_{0}}^{2}$ : environmental variance for production potential, $\sigma_{E_{R}}^{2}$ : environmental variance for resilience, $\rho_{A_{0}A_{R}}$ : genetic correlation, *F*_*N*_: number of flocks, *ϵ*: relative range of environmental challenge levels within each flock compared to the entire range of environmental conditions.

### 2.2 Simulation of the population

#### 2.2.1 Genome in linkage disequilibrium

A similar approach as in Lee and van der Werf (2006); Sánchez-Mayor et al. (2022) was adopted: first, a founder population with its genome in linkage disequilibrium (LD) was simulated using a mutation-drift algorithm (Meuwissen et al., 2001). In this regard, a population with a genome divided in several chromosomes is allowed to evolve. Mutations appear, and drift causes them to be lost or to increase in frequency, and after many generations the genome reaches an equilibrium with segregating SNP at a specific LD pattern determined by the parameters used in the simulation, such as population size and mutation rate. To simulate the pattern of a typical farmed sheep population, the initial population was assumed to have 100 individuals (half males and half females) which were allowed to reproduce for 10,000 generations. Their genome was composed of 26 autosomal chromosomes, each of one Morgan length with 200,000 biallelic loci located equidistantly with a mutation rate of 10^−5^. The mutation rate was tuned such that the LD profile of the final generation matched that of real data for a sheep breed (Kijas et al., 2014). Then, the final generation was expanded within 5 generations to a larger population which served as the gene-pool for sampling the base population for each replicate. The final expanded founder population has 10,000 individuals, 2 haplotypes per individual and per chromosome, 26 chromosomes, and an average number of 4,800 segregating SNPs per chromosome. Hence, for a given replicate, the genome of the base generation was simulated by randomly sampling haplotypes from the expanded founder population; and thereafter the genome of animals from further generations were sampled by dropping haplotypes from offspring to parents assuming Mendelian inheritance law. Two scenarios for pedigree structure and base population was considered as discussed in Section 2.4. Base populations with size 1,200 (1,100 females and 100 males) and 2,970 (2,700 females and 270 males) were simulated from the founder population. In simulation of *N* individuals of base population from the founder population (*N* either 1,200 or 3,970), 2*N* haplotypes were randomly drawn from the pool of 20,000 haplotypes. In addition, sampling of haplotypes was done independently for each chromosome to ensure independency of replicates. As shown in the Supplementary Materials, the probability that two drawn samples are dependent is very small. Moreover, as discussed in the next section, location of SNPs and QTLs as well as their effect was independently sampled for each replicate.

### 2.2.2 Genetic architecture

Once the base generation was sampled in each replicate, 1,500 segregating loci with the highest minimum allele frequency were selected for each chromosome: 500 being randomly assigned to be quantitative trait locus (QTL) affecting the traits and 1,000 being part of the SNP chip array used to calculate the genomic relationship matrix (GRM) needed in the genomic evaluation. The total number of QTLs and SNPs across the whole genome were 13,000 and 26,000, respectively.

The true breeding values (TBV) were calculated as the sum of all QTL effects given the individual’s genotypes. The QTLs were assumed to have pleiotropic additive effects on both the production potential and the resilience traits (*A*_0_ and *A*_*R*_) to allow for non-zero genetic correlation between both traits. The QTL effects for *A*_0_ and *A*_*R*_ were sampled from a bivariate normal distribution with a unit standard deviation and a correlation equal to the targeted genetic correlation between *A*_0_ and *A*_*R*_ (Wientjes et al., 2017), and then they were re-scaled so that the TBV variances for *A*_0_ and *A*_*R*_ in the base generation were equal to the targeted genetic variances. Finally, the environmental deviations *E*_0_ and *E*_*R*_ were sampled to simulate the individuals’ phenotypic performance (using Eq. 1) given the level of exposed environmental challenge at the time of phenotype measurement.

### 2.2.3 Level of environmental challenge

To simulate the level of environmental challenge (*X*), individuals were assumed to be phenotyped in flocks. These flocks represent micro-environments, defined by a range of environmental challenge levels around a flock specific average challenge level. Thus, for an overall environmental challenge level [0, *X*_max_], individuals in a given flock *f* were exposed to a subset ranging between $\left({\hat{X}}_{f}-X_{\max}\frac{\epsilon }{2}\right)$ and $\left({\hat{X}}_{f}+X_{\max}\frac{\epsilon }{2}\right)$ , where ${\hat{X}}_{f}$ is the average challenge effect of flock *f* and *ϵ* is a parameter determining the heterogeneity of the flock conditions. If multiple records were required for an animal, multiple environmental challenge levels were sampled from the same flock.

The simulation of the level of environmental challenge related to a given recorded phenotype was done as follows: firstly, the average challenge level $\left({\hat{X}}_{f}\right)$ for each flock (*f*) was sampled from a uniform distribution within $\left[0+X_{\max}\frac{\epsilon }{2},X_{\max}-X_{\max}\frac{\epsilon }{2}\right]$ ; secondly, each individual was allocated to a given flock and all their phenotypic records were assumed to be recorded in it; third the challenge level *X* for a given phenotype was sampled from a uniform distribution within the range $\left[{\hat{X}}_{f}-X_{\max}\frac{\epsilon }{2},{\hat{X}}_{f}+X_{\max}\frac{\epsilon }{2}\right]$ , and this value was used to calculate the phenotypic performance using Eq. 1. Hence, the level of the exposed environmental challenge is different for all individuals (and among records if an individual has more than one recorded phenotype) but more alike between individuals allocated to the same flock. In this study *X*_max_ was set to 2, which corresponded to an average difference of 6 phenotypic standard deviations in performance compared to the average performance at optimum condition (*X* = 0) (Table 1). Having a relatively large environmental condition allows to define flocks within where we can investigate the effect of, e.g., heterogeneity of flocks and unknown environmental challenge levels.

In this study, ten flocks were simulated, all having equal within-flock heterogeneity, *ϵ*. The magnitude of *ϵ* was set to 0.1, but other values were simulated (Table 1) to assess its impact on the genomic prediction with the RN approach when the environmental challenge level is unknown (see below).

Additionally, several methods for allocating individuals across flocks were used to assess the impact of the distribution of phenotypes across environments: (i) random (RND), where individuals were randomly distributed across all flocks, (ii) clustered (CLS), where individuals from the same half-sib family were allocated into one random flock, (iii) assortative (AST), where individuals from the same half-sib family were allocated to the same flock such that offspring from a sire with the highest breeding values for production potential were allocated to the flock with the lowest level of environmental challenge (i.e., the genetically best animals were allocated to the best environment), and (iv) disassortative (DIS), i.e., similar to AST scenario but with reverse order of sires.

### 2.3 Genetic evaluation

Genetic evaluations were carried out using the best linear unbiased predictor (BLUP) or genomic BLUP (GBLUP) (Meuwissen et al., 2001) defined by the relationship matrix used in the analysis (i.e., the NRM based on pedigree information for BLUP, and the GRM based on genotype information with GBLUP). The breeding values for production potential and resilience were estimated using random regression reaction norm models (Eq. 1) under two scenarios with respect to environmental challenge level: known or unknown.

When the environmental challenge levels were unknown, the genetic evaluation was carried out in a two-step RN approach (Calus et al., 2002; Kolmodin et al., 2002): initially, a genetic evaluation ignoring resilience was performed to estimate the flock effects, and subsequently a random regression was performed using the obtained flock effect estimates as proxy for the challenge level (Kolmodin et al., 2002; Mulder and Rashidi, 2017; Carvalheiro et al., 2019). To calculate the proxy, the estimated flock effect was rescaled such that the flock with highest average performance was assigned as having no challenge (i.e., $\hat{X}=0$ ) and the lowest flock average is assigned the maximum challenge. This proxy value was then assumed to be the challenge level for all phenotypes recorded in the flock in question.

Hence, the use of the proxy with the RN approach introduces two sources of error into the analysis: (i) error in the estimates of the flock effects in the first step of the evaluation, and (ii) error by assigning the same environmental challenge level to all animals in a flock, thus ignoring the within flock heterogeneity in challenge. Therefore, as flocks become more heterogeneous, the estimated proxy becomes more uncertain and less representative of the true challenge level, potentially affecting the quality of the estimates.

In addition, for the comparison of responses to selection based on different breeding criteria traits, EBVs for production performance were calculated using a conventional model without the term associated to the environmental challenge (i.e., the resilience trait) but accounting for the environmental flock effects,

$$y_{jl}=\hat{\mu }+\hat{F_{l}}+\hat{A_{j}}+\hat{E_{j}},$$ (5)

where $\hat{F_{l}}$ is the fixed effect of the flock *l* that animal *j* belongs to, $\hat{\mu }$ is the estimated population average of the production, $\hat{A_{j}}$ is the estimated breeding value of the animal *j* and $\hat{E_{j}}$ is the random environmental effect.

An in-house software was used to estimate the variance components and (G)EBVs using restricted residual maximum likelihood (Patterson and Thompson, 1971; Lee and van der Werf, 2006) and BLUP (Henderson, 1975; Gumedze and Dunne, 2011), respectively. The software is available on https://github.com/mghaderizefreh/GenEval. The genomic relationship matrix was calculated following (VanRaden, 2008). The genetic correlation between production potential and resilience was estimated even when the true genetic correlation was zero.

The quality of the genetic evaluation was assessed by the accuracy and bias of (genomic) estimated breeding values, (G) EBV. The prediction accuracy is defined as the correlation between (G) EBV and the TBV. The bias $\left(\tilde{b}\right)$ is defined as the standardised regression coefficient of TBV on EBV as explained in (Lipschutz-Powell et al., 2012),

$$\tilde{b}=\left\{\begin{array}{cc} 1/b-1\; & b\geq 1\\ b-1\; & b<1 \end{array}\right.,$$ (6)

where *b* is the regression coefficient between (G)EBVs and TBVs. Hence, unbiased estimation has a zero value for the standardised regression coefficient, whereas overestimated or underestimated (G)EBVs have positive or negative standardised coefficients, respectively.

### 2.4 Scenarios compared

As mentioned above, two different population structures were considered to achieve the objectives of this study: To assess the impact of genomics and other factors on prediction accuracies and bias, a sheep population undergoing random selection was simulated with 3 generations and ∼1,000 animals per generation. To assess response to selection, a population undergoing selection for 10 generations under different selection strategies was simulated. The number of animals per generation in the second population was ∼3,000.

Table 1 shows the values of parameters used in the simulations that were common for both populations. For the genetic correlations between production potential and resilience, three values were assumed corresponding to a favourable, zero and antagonistic relationship between the traits. Results are mostly shown for the case of negative genetic correlation as the worst-case scenario, because it has the lowest heritability for production performance across environments (c.f. Eq. 4). Results corresponding to medium and high heritability are provided in of the Supplementary Table S1A.

#### 2.4.1 Population under random selection

The scenarios considered here aim to assess the benefit of genomic prediction in improving the quality of the prediction of production potential and resilience as well as to assess factors affecting the predictions. The population consisted of three non-overlapping generations with a half-sib structure. The base or first generation started with 100 males and 1,100 females, with each male being mated to 11 females to produce one offspring per female, resulting in one male and 10 female offspring (i.e., 1,100 individuals in the second generation, 100 males and 1,000 females). In the second generation, each sire was mated with 10 dams to produce 1,000 individuals in the third generation.

This population was used to assess the impact of different factors on the benefit of genomic prediction to evaluate production potential and resilience. They include:

• The effect of population parameters on the benefit of GBLUP over BLUP in terms of improved accuracy.
• The effect of the distribution of individuals across environments when comparing the GEBV accuracies obtained from individuals across flocks allocated assuming the RND, CLS and AST scenarios.
• The effect of uncertainty of the environment challenge level in a RN model situation by studying the impact of the within-flock level heterogeneity on the range of environmental challenge.

Further, we assume that the third generation does not have phenotypes and we assess the (G)EBVs for this generation only. We use these assumption in order to have generally low accuracy so that the above effects are more pronounced.

#### 2.4.2 Population undergoing multiple generations of selection

Performance records in this section were simulated assuming a genetic correlation of −0.5 between resilience and production potential, with flocks covering 10% of the whole environment and one record for each individual in a random distribution scenario. This population structure was used in scenarios that quantified the selection response for production potential and resilience when using a RN model. The population attempted to mimic a sheep population with large half-sib and small full-sib families. The population structure for each generation consisted of 8,100 individuals (2,700 males and 5,400 females), all genotyped with the SNP chip array and having single phenotypic record. At each generation, half of the females and a tenth of males were selected to mate at random (10 females per 1 male) and a litter size of 3 including 1 male and 2 females was assumed. The assumption of discrete generations and litter size of 3, albeit not very common, (Kenyon et al., 2019), was made to maintain the population size across different generation while allowing for a selection intensity for the females. Genetic evaluations were performed using either the RN approach (with unknown environmental challenge level) or the conventional method without RN (i.e., using Eq. 5).

When using RN approach, a selection index was defined as $I=\left(1-\alpha \right)\hat{A_{0}}+\alpha \hat{A_{R}}$ where *α* (0 ≤ *α* ≤ 1) is the weight given to resilience. Hence, *α* = 0 corresponds to selection for production potential only, whereas *α* = 1 corresponds to selection for resilience only. The selection scheme was done over 10 discrete generations and individuals were allocated using the RND allocation scenario. We also contrasted scenarios where performance records were generated in a narrow compared to a wide range of environments (Table 1).

## 3 Results

### 3.1 Random selection scenario

#### 3.1.1 Comparison between pedigree based and genomic predictions

**FIGURE 1:**
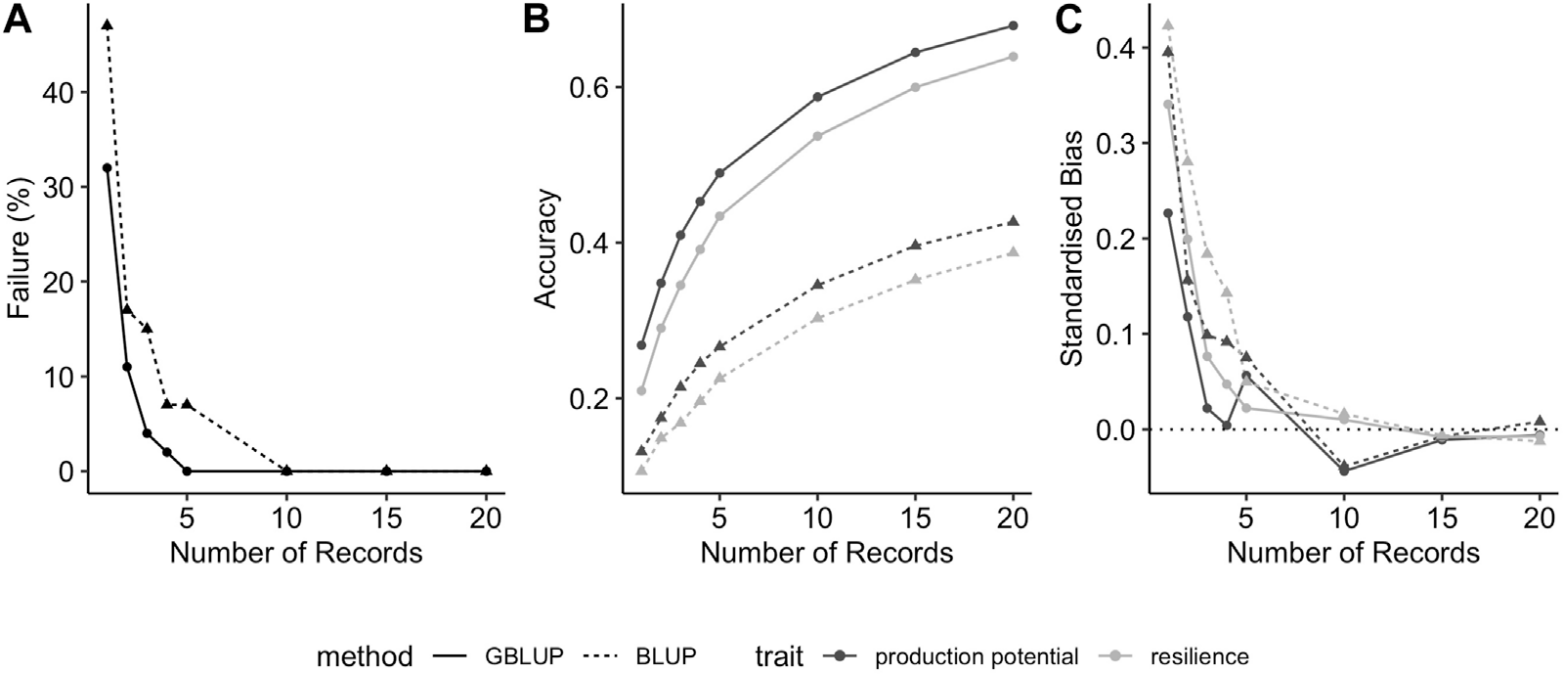
Effect of number of records per individual on **(A)** percentage of simulations where REML failed to converge to feasible variance components **(B)** prediction accuracy of production potential and resilience, and **(C)** standardised bias in EBV for production potential and resilience, when using GBLUP (solid lines and circles) or BLUP (dashed lines and triangles).

Figure 1 shows the BLUP and GBLUP results for the genetic evaluation of production potential and resilience depending on the number of phenotypic performance records available per individual. As expected, the extra information from increasing the number of records per individual resulted in better estimation leading to less convergence failures in the REML analysis (Figure 1A), and higher GEBVs accuracies (Figure 1B) with lower standardised biases (Figure 1C) in the GBLUP/BLUP analyses. The average prediction accuracies of EBVs from BLUP for production potential and resilience incremented as much as 70% when the number of records per individual was increased from one to three (i.e., the accuracies for production potential and resilience with one record per individual were 0.13 and 0.10, respectively, compared to 0.22 and 0.17 with three records per individual). Consistently across the scenarios, the GBLUP evaluation outperformed BLUP. For instance, when assuming three records for each animal, the REML analysis using pedigree information failed to converge in 15% of the replicas whereas using genomic information only 4% of the replicates failed to converge (Figure 1A). For the same scenario the GEBV accuracy was 91% and 105% higher than that of EBVs for production potential and resilience, respectively (Figure 1B). In particular, for both traits, prediction accuracy assuming one record per animals with GBLUP was as high as that of BLUP with five records. Regardless of the method used (pedigree or genomic), estimated breeding values for production potential were in general more accurate than those for resilience, e.g., for three records per individuals, prediction accuracies for production potential were 19% higher than those for resilience. Some degree of bias in the estimates were observed in scenarios considering one or two records available per individual, but this tended to be slightly less on estimates from GBLUP than with those estimates obtained using BLUP (Figure 1C). Since the frequency of variance component estimation giving non-zero estimates was lower for *n* = 3, all our comparison based on this pedigree structure were done assuming 3 records per individual.

**FIGURE 2:**
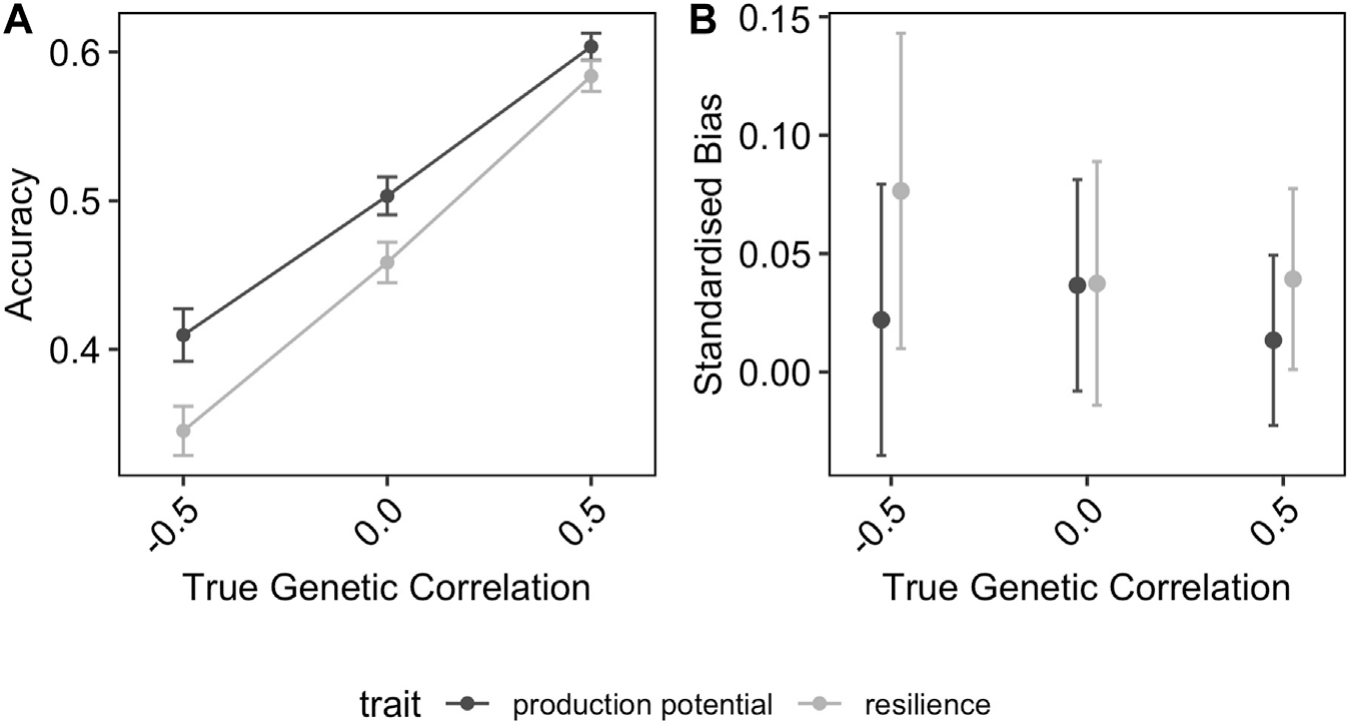
Effect of true genetic correlation between production potential and resilience on **(A)** the prediction accuracy of GEBVs and **(B)** the standardised bias in GEBV (positive values correspond to over-estimated GEBVs). The error-bars are the 95% confidence intervals.

Moreover, the observed trends were consistent across different genetic correlations between production potential and resilience and between BLUP and GBLUP (results not shown). In particular, as would be expected, prediction accuracies for both traits increase with increasing genetic correlations (as shown for GEBVs in Figure 2A): the average prediction accuracies of production potential and resilience increases from 0.41 to 0.60, and from 0.34 to 0.58, respectively. In addition, the resilience tends to be upward biased, and this intensifies when the genetic correlation is negative (Figure 2B).

For the remainder of this section (Random selection scenario), we will only show results for GEBVs assuming three records per individual and genetic correlation of −0.5 between production potential and resilience. This scenario was chosen to show how potential trade-offs between the traits may affect prediction accuracies, and because it has a low rate of failure to converge in the REML estimation ensuring that the results are true reflection of the situations compared and not to a lack of information due to inadequate population size. Furthermore, the observed effects of distribution of phenotypes across environments and uncertainty in the level of environmental challenge described below were similar for EBVs and GEBVs.

#### 3.1.2 Effect of distribution of phenotypes across environments

**FIGURE 3:**
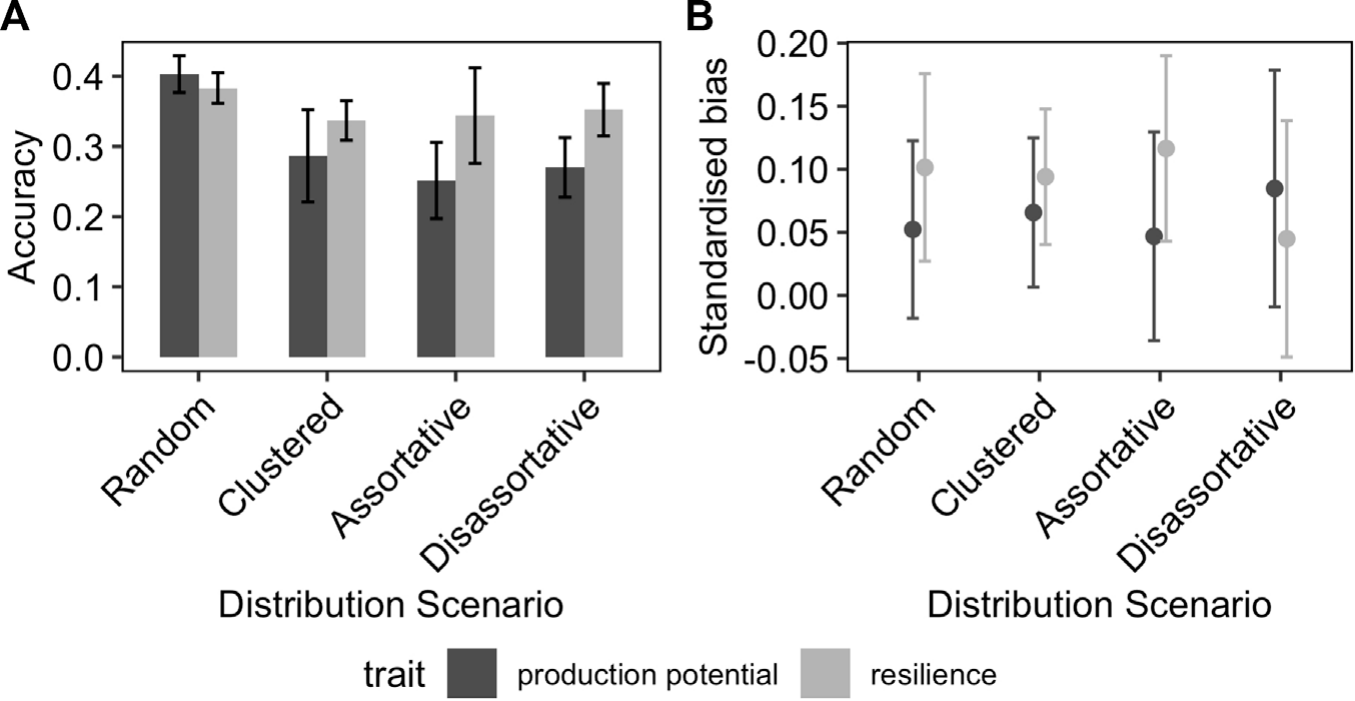
Effect of distribution of phenotyped animals on the **(A)** prediction accuracy and **(B)** standardised bias of GEBVs. The error-bars are the 95% confidence intervals.

Figure 3 shows the effect of the distribution of phenotyped animals across environments with different average challenge levels on the accuracy and the bias of the GEBV for resilience and production potential. In general, the distribution of phenotypes across environments affected the quality of the genetic prediction of both traits but in different ways. The accuracy of the production potential was affected by the distribution of phenotypes across environments, with the highest accuracy (0.41) observed in the RND distribution scenarios and the lowest in AST and DIS scenarios (0.32). This was not the case for the resilience GEBVs, to which their accuracies were almost identical for the different allocation scenarios. In particular, even though the accuracy of production potential is generally higher than that of resilience, in the scenarios AST and DIS, resilience is estimated on average more accurately than the production potential. On the other hand, the distribution of phenotypes across different environments affected the resilience GEBV mainly, on the degree of bias, with the AST scenarios introducing the highest standardised bias of 15% (Figure 3B).

#### 3.1.3 Effect of uncertainty in the level of environmental challenge

**FIGURE 4:**
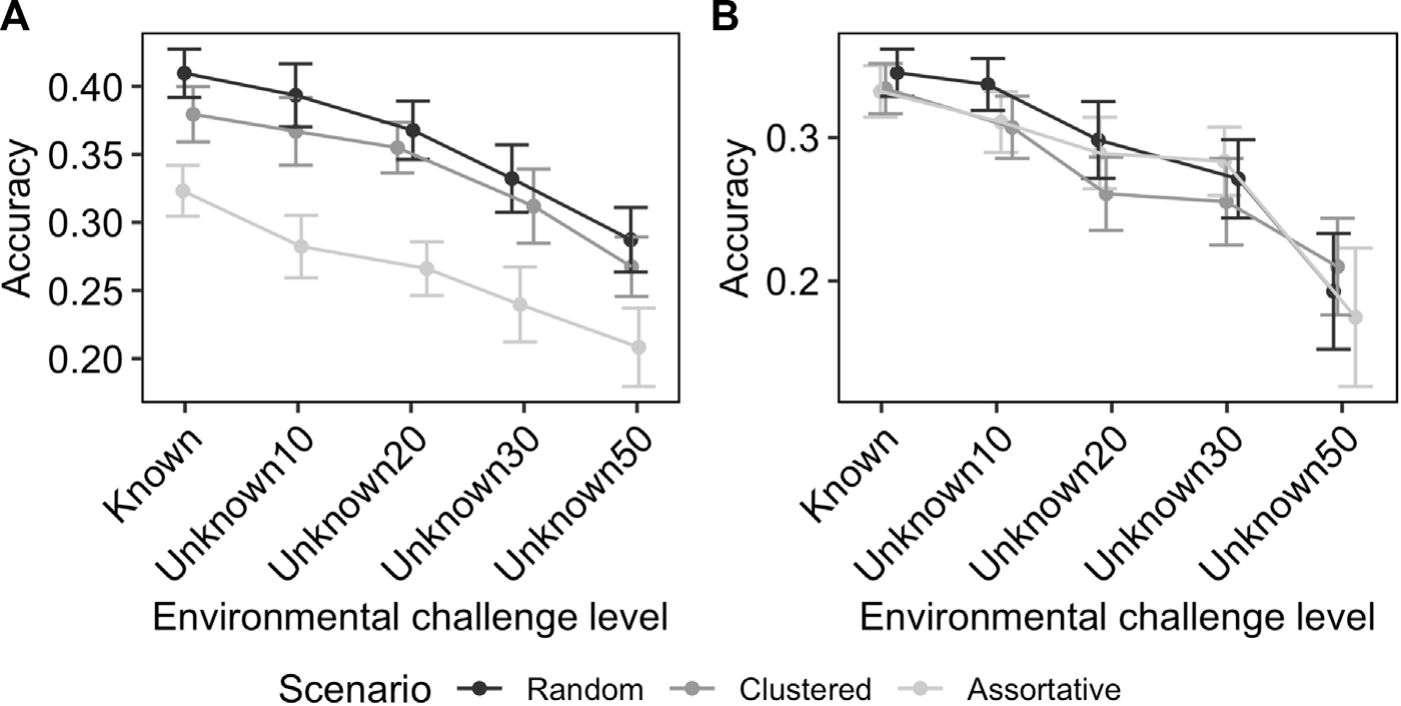
Effect of unknown environmental challenge in RN model on the accuracy of GEBV for production potential **(A)** and resilience **(B)**. The numbers after the word “Unknown” on the *x*-axis indicate the heterogeneity of flocks in terms of percentile coverage of the entire range of environmental conditions. The effect is shown for different distribution scenarios with different colours. The error-bars indicate the 95% confidence intervals.

Figure 4 shows the effect of uncertainty in the knowledge of challenge level on the GEBV accuracies. In the ideal scenario, where the challenge levels are known without error, the GEBV accuracy was 0.41 for production potential and 0.35 for resilience. When the heterogeneity of flocks was 10%, as modelled here by flocks exposed to larger ranges of environmental challenge levels with average performance used as a proxy, the reduction on the GEBV accuracy was small (e.g., for RND scenario the average prediction accuracy for resilience and production potential changes from 0.44 to 0.49 to 0.46 and 0.50, respectively). However, as the level of uncertainty increased the GEBV accuracy further decreased, to the level that for the scenario Unkown50 (where each flock covered up-to 50% of full environmental challenge range) the GEBV accuracy was 0.28 and 0.21 for production potential and resilience, respectively. The rate of reduction in GEBV accuracy with increasing uncertainty was similar for different distribution scenarios (Figure 4).

### 3.2 Directional selection scenario

**FIGURE 5:**
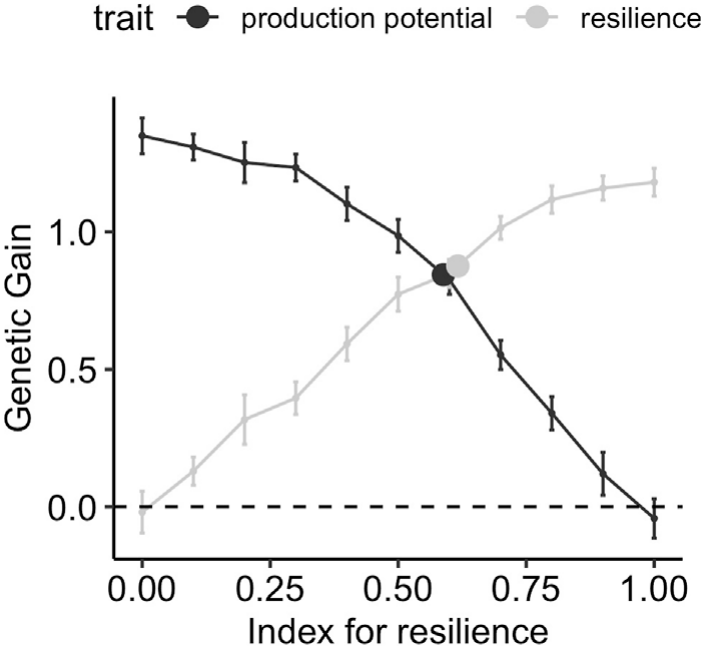
Genetic gain after 10 generations of selection using different strategies, when performance records are obtained from a wide range of environmental challenge levels ([0,2]). The lines correspond to using a selection index comprising production potential and resilience with different relative weights on resilience on the *x*-axis, and points correspond to selection on production performance GEBVs obtained using the conventional model (Eq. 5)

Figure 5 shows the gain in production potential and resilience after ten generations of selection when phenotypes are collected from a wide range of environmental challenge levels, spanning over six phenotypic standard deviations in average performance. The genetic gain is shown for different weights for resilience in the selection index. The response to selection for the two traits when the conventional model for performance (i.e., ignoring the environmental challenge and only fitting the flock as fixed effect) (Eq. 5) is used, is indicated with a point and is moved horizontally to lie on the selection index profile. Figure 5 shows a smooth transition of the genetic gain for resilience from negative to positive values as the index for the resilience is varied from 0 to 1, and the reverse is true for production potential. The maximum achievable relative gains for production potential and resilience using corresponding indices are 1.4 and 1.3, respectively. This means that using an index with all weights on production potential, the average production potential is increased from 10 to 11.4 after 10 generations. Similarly, *α* = 1 in the selection index increases the resilience by 43.3% from −3 to −1.7 after 10 generations (cf. Table 1). The genetic gain from the conventional model indicates that in such a scenario both resilience and production potential can be improved simultaneously with a relative gain of 0.78 for both traits, equivalent to an index with weights 0.58 and 0.42 for resilience and production potential, respectively.

**FIGURE 6:**
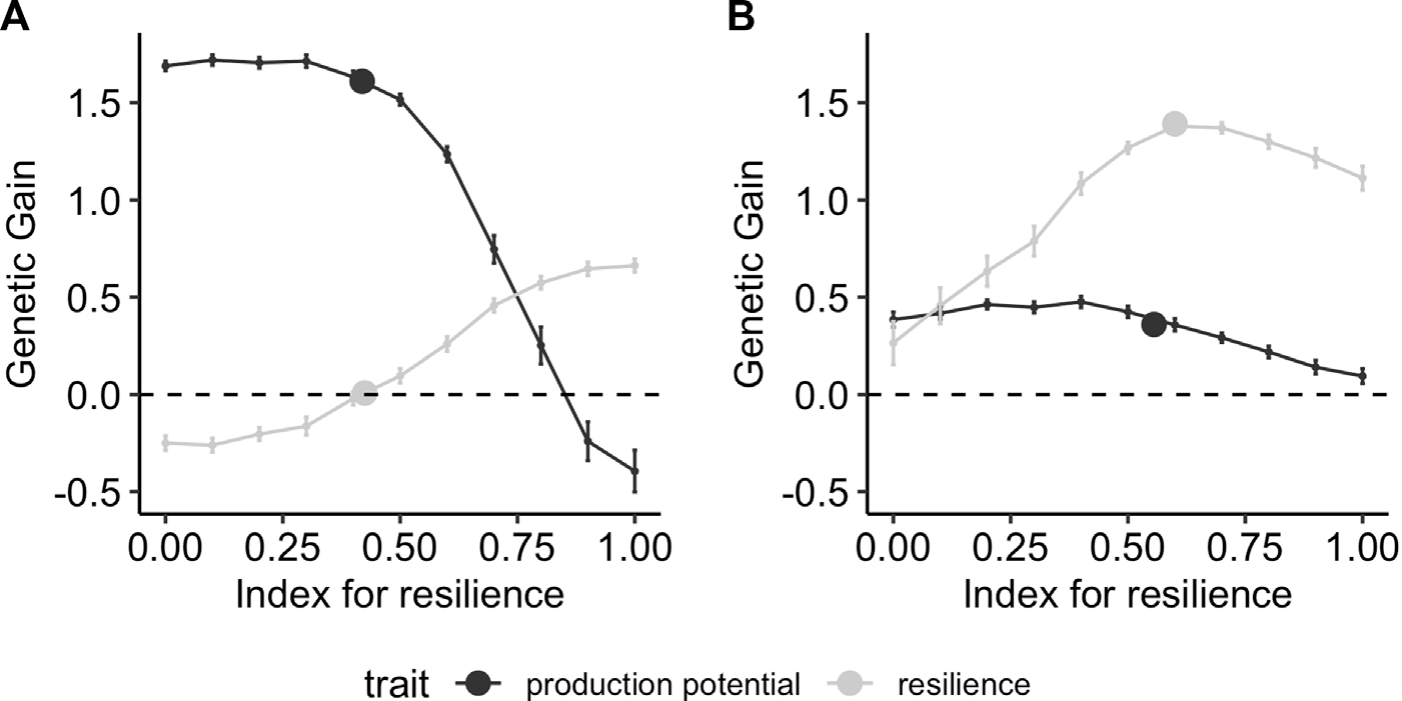
Genetic gain after 10 generations of selection using different strategies on performance records obtained in narrow environmental conditions with mainly **(A)** low (*X* ∈[0,1]) and **(B)** high (*X* ∈[1,2]) challenge levels. The lines correspond to using a selection index comprising production potential and resilience, with different relative weights on resilience on the *x*-axis, and the points correspond to selection on production performance GEBVs obtained using the conventional model (Eq. 5).

The response to selection after 10 generations of selection depends strongly on the range of environments in which phenotypes are collected. Figure 6 shows the results when phenotypes are collected in a narrow range of good or bad environmental conditions, corresponding to ranges of [0,1] and [1,2], respectively. In good environmental conditions (i.e., low challenge levels, Figure 6A), the maximum achievable gain for resilience (i.e., an index with all weights on resilience) is 0.7 whereas the maximum relative genetic gain in production potential (i.e., an index with all weight on production potential) is up to 1.6. Using a conventional model when phenotypes are mainly collected from a narrow range of environmental conditions with low challenge, only production potential is improved, whereas the relative genetic gain in resilience is zero. Conversely, collecting data mainly from bad environments (Figure 6B) results in higher gains in resilience. The maximum available gain for production potential with a selection index reduces to 0.5 in this case (i.e., one-third of what is observed when phenotypes are collected in good environments), whereas that of resilience increases to 1.4 (i.e., twice as much as when phenotypes are from good environments). Similarly, the genetic gain that can be achieved when analysing phenotypes collected in bad environments with the conventional model results in improvement of resilience mainly, with a relative gain of 1.5 compared to the 0.35 gain for production potential. It is also noteworthy that the conventional model yields close to maximum genetic gain in resilience if phenotypic records are collected under high challenge conditions, whereas it leads to zero improvement of resilience when only phenotypic records in low challenge levels are available. In the latter case, genetic gain in resilience can only be achieved using reaction-norm models.

## 4 Discussion

In this study, we demonstrated the benefit of genomic prediction on the accuracy and bias of the estimated breeding value of resilience and production potential traits for data simulated with RN models. We also showed that the number of phenotypic records, the distribution of phenotypes across different environments, and the degree of uncertainty on the challenge levels can affect GEBV accuracy and bias. However, even for sub-optimal distribution of phenotypes or highly heterogeneous and unknown challenge levels, accuracies are sufficiently high, and bias is sufficiently low to select simultaneously for both traits.

It has been widely reported in literature that the use of genomic information improves the accuracy of breeding values in genetic evaluation for different species (VanRaden et al., 2009; Riggio et al., 2014; Wolc et al., 2016; Tsai et al., 2016; Sánchez-Mayor et al., 2022). Evidence on the beneficial effect of genomic information when using a RN model is also available from real data. For example, Silva et al. (2014) reported that the GEBVs for total number of born in pigs at a given environment were about 70% more accurate than EBV accuracy obtained using pedigree information. Moreover, recently it has been shown that ssGBLUP implementing a RN model on production traits in pigs yields prediction which were 10 ∼25% more accurate than a RN using pedigree alone (Fragomeni et al., 2016; Song et al., 2020). Whilst these studies estimated GEBVs for production potential and resilience, their criterion of comparison was based on the accuracy to predict performance at a given environment with a specific level of challenge. The emphasis in this study, however, was to evaluate the benefits of using genomic information on prediction accuracies for the two traits, production potential and resilience, themselves.

We observed that the benefit of GBLUP in increasing GEBV accuracies was greater for resilience than for production potential, which is partly explained by the fact that the resilience trait has lower accuracy from the start. It is important to note that the beneficial effect of GBLUP over BLUP was the same regardless of the number of phenotypic records available per individual. Hence, one may extrapolate that similar benefits of using genomic information should be expected in other populations that may differ in size or the number of phenotypic records available.

Whilst the GEBV accuracy for production potential was generally greater than for resilience, production potential GEBVs were more sensitive to the distribution of animals across environments, compared to the resilience trait, whose accuracy remained barely unchanged across the different scenarios on how the phenotypes are distributed across the environments. It is important to note that for the extreme case of AST scenario, the production potential GEBVs was severely affected to the point that their accuracy was on average lower than that for resilience. It is known that accuracy of EBVs for production potential (estimated with pedigree) are lowest when animals are reared in clusters and the correlation between the exposed challenge level and the production level is negative (i.e., the AST scenario) (Calus et al., 2004). The most plausible explanation for the lower accuracy in the AST scenario may be due to genetic and environment factors being confounded and that genetic connectedness is reduced between herds (Foulley and Quaas, 1995). We observed that this trend remains true also for GEBVs. For a BLUP evaluation, information of EBV for an individual comes from its own performance plus that of closely related individuals, so clustering them in environments with similar challenge level would be expected to affect EBV accuracy (especially for AST scenario where the best families are reared in the best environment). However, for GBLUP evaluation, the GEBVs for a given individual are based on their own information and from all recorded individuals, related or unrelated; hence one may expect that GEBV accuracies being less sensitive to the distribution of phenotypes. However, our results show that this is not the case, and the use of genomic information would not overcome the negative impact of member of the same family being evaluated in similar environments as in CLS or AST. Note that although the GEBV accuracy for resilience is relatively unaffected by the system of recording performance across the environment, some degree of bias in the resilience estimate tend to appears as the scenario become more like the CLS or AST. Therefore, for estimating both the production potential and resilience, it is important that the phenotypes from related individuals are collected from different environmental conditions as was represented by the RND scenario in this study. Unfortunately, the pattern of distribution of phenotype across environments is heavily influenced by the production system. Populations where natural mating is common would lead to scenarios similar to AST or CLS, but the widespread use of artificial insemination may allow for a more mixing of families across environments. Unfortunately, artificial insemination has been proved to be challenging for some species like sheep (Carta et al., 2009). Additionally, scenarios with large use of artificial insemination may also lead to clustered distribution of phenotypes, where good farms with high management input may result in environments with low challenge but also having the resources to buy the semen from the best sires (hence resulting in AST situation). The results from our study further highlight the need to promote as much as possible an un-clustered distribution scenario if resilience and production potential are to be accurately evaluated without bias. Based on our results, as shown in Figure 3, for breeding values estimated with GBLUP, the use of genomic evaluation would not counterbalance this detrimental clustering effect and the accuracies are lower when animals are clustered.

The RN models are the most common approach in disentangling the environmental effect from the genetic effect on the phenotype (Calus et al., 2004; Mulder, 2016). In many real-world scenarios, the environmental challenge is unknown or simply not measured. In order to fit random effects for the level of response to environment, i.e., the resilience, a proxy is estimated for the challenge level using the same data to be analysed. The drawback is that this approach requires to separate performance into discrete classes (e.g., contemporary groups), where all individuals in each class are assumed to be affected by the same challenge level, so the genetic evaluation is done assigning the same proxy value to all individuals in the class. We show that accuracy of GEBVs obtained from this approach are not compromised if the discrete classes are relatively homogeneous, i.e., cover a small range of environments. This is likely not to be the case, particularly for infectious diseases, where exposure to infectious pathogens is highly stochastic in nature (Bishop and Woolliams, 2010). The error in the assigned challenge level would be expected to affect the accuracy of the GEBVs. Furthermore, the magnitude of this detrimental effect is expected to be related to the within-group heterogeneity in the challenge level. Our results showed that the loss in accuracy due to uncertainty on the level of the challenge should be minimal, if classes to calculate the proxy are created such as that their heterogeneity within (defined as the range of possible environmental challenge of within the class, relative to the whole) is under 10%. However, although the large impact that uncertainty on the challenge level can have on the quality of the prediction, our results showed that relatively good genetic estimates can still be achieved even when these environments are very heterogeneous (e.g., 50%). Whilst the implementation of an RN evaluation should have extra care in the allocation of performance records in discrete classes, (to reduce environmental heterogeneity within class), we have shown that even when these classes are very heterogeneous, the GEBV can be of sufficient accuracy so their use can result in successful genetic progress in practical breeding programmes.

Over the recent decades, the animal breeding community has given much attention to breeding for resilience. However, it is desirable that by selecting for increased resilience, one does not accidentally select for poor performers when conditions are good. It has been postulated (Falconer and Mackay, 1996), and shown here, that selecting for animals that perform well in bad environments increases resilience when a conventional model is used without RN. However, this may result in little gain for the production potential if not a decrease when the genetic correlation between production potential and resilience is negative. Improving both traits simultaneously is easier when there is no trade-off between traits (Mulder, 2016). However, many studies report an antagonistic genetic correlation between resilience and production potential (Carabaño et al., 2017; Sánchez-Molano et al., 2020; Freitas et al., 2021). Additionally, it has been shown, using a more complex model, that the estimates of heritability are smaller when animals are exposed to micro-environmental disturbances, *X*_*d*_ > 0, compared to an environment with no disturbance, *X*_*wd*_ = 0, (Le et al., 2022). This translates to heritability of performance at *X*_*d*_, i.e., *h* ^2^ (*X*_*d*_), being smaller than heritability at *X*_*wd*_, which is *h* ^2^ (*X*_*wd*_) = *h* ^2^ (0) in Equation 4, which occurs always for negative genetic correlation and small *X*_*wd*_.

In this study, linear reaction models were used. Changes in performance of animals may however not always be linear with respect to challenge level. Therefore, non-linear reaction norms (e.g., quadratic) can predict phenotypes at specific environments more accurately Pollott and Greeff (2004); Sánchez-Molano et al. (2020). To improve resilience with such models a more complex selection index may be needed because all the coefficients from the model contribute to the sensitivity of an animal to a challenge and hence need to be incorporated in the selection index. Furthermore, in our study, we used a linear heterogeneous residual term and continuous challenge level. Heterogeneous residual variances can be modelled with exponential function (Hill and Mulder, 2010), as is the case in uniformity studies. While the definition of the heritability for resilience is more straightforward with our model, it is important that the conclusions drawn in this study are tested in other scenarios.

Lastly, in this paper, we defined resilience as the ability of an animal to maintain high production performance when exposed to challenge, in line with Bisset and Morris (1996). Resilience was simulated as a reaction norm, where one performance record for an animal corresponds to a given environmental challenge, *X*. This approach is valid when there is a relatively constant challenge level over a significant period of animals’ life, e.g., prolonged nutritional shortage or heat stress, and when the effects on the animal phenotype of interest can be captured by a constant value, e.g., growth rate over a specific time period, or carcass weight. Several more recent studies, including the EU Horizon 2020 Smarter consortium (SMARTER, 2018) define resilience as the ability of an animal to either maintain or revert quickly to high production or health status when exposed to challenge or micro-environmental disturbances (Colditz and Hine, 2016; Berghof et al., 2019; Le et al., 2022). To estimate resilience according to this refined definition of resilience, longitudinal performance measures of individual animals would be required to capture, e.g., ability of the animal to return to its pre-challenge state after exposed to a challenge (Colditz and Hine, 2016; Berghof et al., 2019; Knap and Doeschl-Wilson, 2020). Thanks to the automated phenotype measurement devices that the animal industry is adopting, daily measurements of performances are becoming more available (Poppe et al., 2020; Neethirajan and Kemp, 2021). Recent studies have produced and evaluated novel resilience indicators from such daily measurements (Putz et al., 2019; Nguyen-Ba et al., 2020; Poppe et al., 2021). For example, Le et al. (2022) defined and used resilience and resistant terms in simulated longitudinal body weight of growing pigs and showed environmental disturbances affect estimates of breeding values. Future studies are warranted to assess the benefits of genomics and the influence of diverse factors as those assessed here, on these novel resilience indicators.

## 5 Conclusion

In this paper, we studied the benefits of using genomic information, random regression RN models and phenotyping strategies on genetic evaluations of resilience and production potential. We showed that simultaneous improvement of both traits is possible even under unknown environmental challenge conditions if genomic information is used in a RN model and phenotyped animals are not in family clusters. In contrast, genetic improvement in resilience without the use of RN models is possible if genotyped and phenotyped animals are reared in wide range of environmental conditions. Therefore, for the best genetic evaluation, use of artificial insemination or other methods that increase connectedness among genotypes in various environmental conditions, is recommended. Additionally, higher between-variance for different environmental conditions facilitates simultaneous genetic improvement for resilience and production potential.

## Data Availability

The raw data supporting the conclusion of this article will be made available by the authors, without undue reservation.
