# Supplementary material for: Exploring the value of genomic predictions to simultaneously improve production potential and resilience of farmed animals
Source: Front Genet. 2023 May 12;14:1127530. doi: 10.3389/fgene.2023.1127530 (PMC10213464; doi:10.3389/fgene.2023.1127530)
Supplement: Supplementary file 1 [file Table1.docx]

Table A.1. Accuracy of Production potential and resilience estimated using BLUP or GBLUP at various heritability (h^2^) and genetic correlations (r). Numbers in parentheses are 95% confidence intervals. Heritability of production potential and resilience are assumed to be equal therefore the value h^2^ corresponds to both. Simulations are done for known challenge level and random distribution of individuals across different herds.

| Trait | method | Variance component | | | | | | | | |
| --- | --- | --- | --- | --- | --- | --- | --- | --- | --- | --- |
|  |  | h^2^ = 0.1  r = -0.5 | h^2^ = 0.1  r = 0.0 | h^2^ = 0.1  r = +0.5 | h^2^ = 0.3  r = -0.5 | h^2^ = 0.3  r = 0.0 | h^2^ = 0.3  r = +0.5 | h^2^ = 0.6  r = -0.5 | h^2^ = 0.6  r = 0.0 | h^2^ = 0.6  r = +0.5 |
| Production potential | BLUP | 0.307  (0.0170) | 0.382 (0.0136) | 0.432 (0.0151) | 0.335 (0.0143) | 0.413 (0.0110) | 0.509 (0.0077) | 0.46 (0.0116) | 0.524 (0.0092) | 0.586 (0.0068) |
| Production potential | GBLUP | 0.408  (0.0177) | 0.470 (0.0185) | 0.520 (0.0131) | 0.441 (0.0159) | 0.523 (0.0119) | 0.631 (0.0073) | 0.585 (0.0129) | 0.650 (0.0102) | 0.719 (0.0058) |
| Resilience | BLUP | 0.283 (0.0145) | 0.351 (0.0175) | 0.428 (0.0134) | 0.316 (0.0109) | 0.391 (0.0096) | 0.491 (0.0098) | 0.441 (0.0094) | 0.494 (0.0076) | 0.572 (0.0063) |
| Resilience | GBLUP | 0.365 (0.0148) | 0.452 (0.0174) | 0.521 (0.0152) | 0.42 (0.0110) | 0.498 (0.0101) | 0.607 (0.0089) | 0.566 (0.0095) | 0.623 (0.0077) | 0.704 (0.0061) |
