# Supplementary material for: Exploring the value of genomic predictions to simultaneously improve production potential and resilience of farmed animals
Source: Front Genet. 2023 May 12;14:1127530. doi: 10.3389/fgene.2023.1127530 (PMC10213464; doi:10.3389/fgene.2023.1127530)
Supplement: Supplementary file 2 [file DataSheet1.docx]

## Independence of replicates of base population sampled from the gene-pool

In order to ensure different replicates are independent several randomisation steps were done from drawing haplotypes for base population to simulation of breeding values. The following shows the probability that two replicate have same haplotypes drawn from the gene-pool.

The probability that two replicates selected without replacement from a total of $N$ objects ($m<N$) and have at least $p$ shared objects ($p\leq m$) can be calculated using the hypergeometric distribution. In our study, $N=2\times10,000$ is the number of haplotypes for the founder population, $m=2\times\left( 2700+270 \right)$ (with 270 males and 2700 females) is the number of haplotypes that is needed to simulate the base population for the larger pedigree, and $p$ is the number of haplotypes that are assumed to be chosen the same in two different replicates.

The number of ways to choose $m$ from $N$ haplotypes in the first replicate is $C\left( N,m \right)=\frac{N!}{m!\left( N-m \right)!}$. There are equal number of ways for the second replicate. Therefore, in total we have $C\left( N,m \right)^{2}$ options. Next, we want to count the number of ways such that $p$ haplotypes in chosen $m$ haplotypes are shared between two replicates. There are $C\left( m,p \right)$ ways that these $p$ haplotypes are in the chosen $m$ haplotype in the first replicate and there are $C\left( N,m \right)$ ways to choose $m$ from $N$ haplotypes. Hence, a total of $C\left( N,m \right)\cdot C\left( m,p \right)$ ways to choose $p$ in $m$ combinations from $N$ haplotypes in the first replicate. Now we want to count the number of ways in the second replicate such that out of combinations with $m$ member, at least $p$ members are chosen that are in the first replicate. We can choose $m-p$ haplotypes from $N-p$ and add the other $p$ haplotypes which would be $C\left( N-p,m-p \right)$. Hence, the final answer for the probability is

$$\frac{\left( C\left( N,m \right)\cdot C\left( m,p \right)C\left( N-p,m-p \right) \right)}{\left( C\left( N,m \right) \right)^{2}}=\frac{C\left( m,p \right)C\left( N-p,m-p \right)}{C\left( N,m \right)}.$$

For example, given a founder population with $N=2\times10,000$ individual haplotypes, choosing $m=2\times\left( 2700+270 \right)$ individual haplotypes, the probability that **at least** 3’000 haplotypes are shared between two replicates is $2.84\times{10}^{-115}$. Using simulations, it can be shown that around 1764 haplotypes (i.e., 30%) will be shared between two replicates for the given number of $N$ and $m$. However, this does not mean that there will be 1764/2=882 identical individuals between two replicates because this calculation was done only for one chromosome. The probability that two base populations from two replicate share the whole genome is ${0.3}^{26}=2.54\cdot{10}^{-14}$. In addition, haplotypes are not sampled in pairs therefore, an individual in the base population may have two haplotypes from two different individuals. Moreover, we sample QTL and SNPs at random location in each replicate and QTL values are also sampled independently for each replicate.

Furthermore, we, empirically, tested the independency between replicates by examining the genetic relationship between pair of replicates. To do so, we sample two replicates from the founder population used in our study and (assuming that the loci used in each replicate is the same, which it was not the case in our study) calculated the GRM for the population composed of the base population for these two replicates. Here, 1200 individuals were simulated for each replicate from a founder population with of size 10,000. Therefore, the combined population is 2,400 individuals. The resulting GRM is

| $G=\left( \begin{matrix} G_{11} & G_{12} \\ G_{12} & G_{22} \end{matrix} \right).$ | A1 |
| --- | --- |

where $G_{11}$ and $G_{22}$ (approximately) are the GRM for base populations 1 and 2, respectively, and $G_{12}$ is the relationship matrix between individuals in replicate 1 and 2. The following histogram shows the values of $G_{12}$. As shown in this Figure A.1, 94% of all possible pairs of individuals have a genetic relationship of less than 0.05. This result indicates that, for our specific population size, the haplotypes sampling resulted in very lowly related individuals, hence the replicates can be considered as independent.


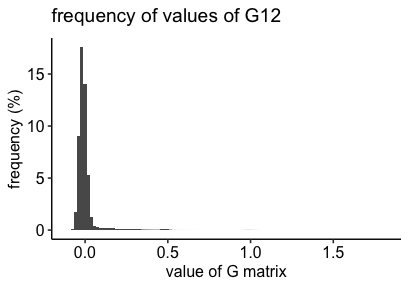


Figure A.1. Frequency of values in the off-diagonal block of Gmatrix ($G_{12}$ in Equation A1) for a population composed of two replicates
